# An Intraoperative Case of Spontaneous Restoration of Circulation from Asystole: A Case of Lazarus Phenomenon

**DOI:** 10.1155/2012/380905

**Published:** 2012-01-24

**Authors:** Konstantinos A. Ekmektzoglou, Eleni Koudouna, Eleni Bassiakou, Konstantinos Stroumpoulis, Phyllis Clouva-Molyvdas, Georgios Troupis, Theodoros Xanthos

**Affiliations:** ^1^MSc Program in “Cardiopulmonary Resuscitation”, School of Medicine, University of Athens, 75 Mikras Asias Street, 11527 Athens, Greece; ^2^Department of Anaesthesiology, “Thriassio” General Hospital of Elefsina, Gennimata Avenue, 19600 Magoula, Greece; ^3^Intensive Care Unit, “Thriassio” General Hospital of Elefsina, Gennimata Avenue, 19600 Magoula, Greece; ^4^Department of Anatomy, School of Medicine, University of Athens, 75 Mikras Asias Street, 11527 Athens, Greece

## Abstract

This case report refers to a victim of intraoperative cardiac arrest, who restored spontaneous circulation despite of cessation of cardiopulmonary resuscitation (CPR). The victim, a 53-year-old man, was undergoing a surgical investigation and rehabilitation of a thigh hematoma. Two minutes after discontinuation of a 46 min CPR, a normotensive sinus node rhythm appeared at monitor. Despite of lack of an adequate explanation, the authors believe that the combination of the high total dose of adrenaline with the cessation of mechanical ventilation might augment venous return and lead to restoration of spontaneous circulation.

## 1. Introduction

The incidence of return of spontaneous circulation (ROSC) after discontinuation of cardiopulmonary resuscitation (CPR) might be one of the strongest evidence of medicine that ignorance is hiding behind myth. This phenomenon was named as Lazarus phenomenon in 1986 [1], although it had been reported for the first time many years earlier by Linko et al. [2]. The occurrence of this phenomenon is suspected to be frequent and underestimated [3].

## 2. Case Presentation

A man was dispatched in the emergency room by the Emergency Medical Service, after self-shooting himself. The patient was immediately transferred to the operating theatre, where he had undergone hemicolectomy, pericardiectomy, splenectomy, and left nephrectomy. After surgery, he was hospitalized in the Intensive Care Unit (ICU).

The weaning of the patient proved to be extremely difficult, leading to a thirty-day hospitalization in ICU; his morbid obesity (160 kg), his depression, and—of course—the complications of his initial critical illness, such as acute renal failure treated with haemodialysis, ischaemic stroke, and multiple infections, led to a prolonged ICU stay.

When attempts were made to cannulate the left femoral vein, a femoral hematoma was developed. Femoral artery rupture was diagnosed by ultrasound. The patient became haemodynamically unstable, while the monitor recorded sinus tachycardia. The patient was immediately transferred to the operating theatre for surgical restoration of the femoral artery. The left femoral venous catheter was removed and replaced by a right femoral venous catheter. A right femoral arterial catheter was also placed, in order to monitor the patient's blood pressure (BP) invasively. Despite continuous administration of fluids and noradrenaline, his BP remained low (BP = 75/32 mmHg).

 Five minutes after the initiation of the surgical procedure, the patient's cardiac rhythm converted from a sinus tachycardia of 165 beats per minute (bpm) to a sinus bradycardia of 48 bpm and his arterial pressure wave decreased and finally disappeared. Cardiac arrest (CA) was diagnosed and the advanced life support algorithm was followed, with chest compressions, ventilation, and drug administration [4]. During CPR, the femoral artery was surgically repaired. Blood transfusion was discontinued when the patient's haemoglobin (Hb) was 10.2 mg/dL. The patient's oxygenation and the electrolytes did not show any abnormal values during resuscitation (Table 1).

**Figure 1:**
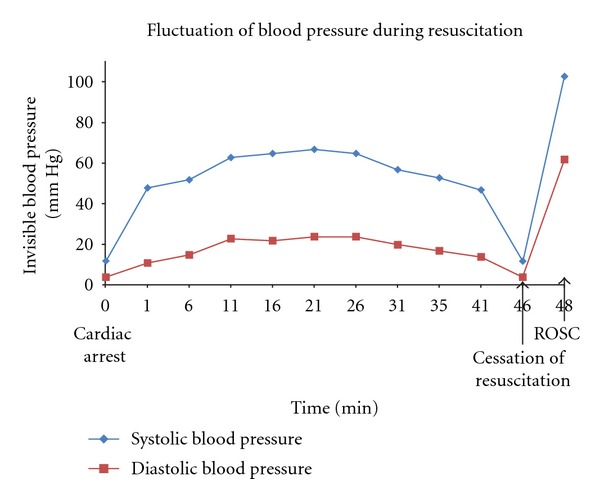
Blood pressure (BP) fluctuation during cardiac arrest in our patient.

The patient's diastolic pressure during chest compression varied from 11 mmHg to 24 mmHg (Figure 1). After 46 min of CPR, the bleeding had stopped, his Hb level had been normalized, and there was no other possible reversible cause of CA. The patient finally developed asystole. Two minutes after cessation of CPR, the rhythm changed to normal sinus (72 bpm) and a normotensive arterial wave (BP = 103/62 mmHg) appeared at the monitor.

No effort of spontaneous breathing was observed. After ROSC, the surgical wound closure continued, the patient was stabilized for 30 min and was transferred to ICU. During the patient's postresuscitation hospitalization in ICU, which lasted for 34 days before dying, his BP was preserved by noradrenaline infusion and no spontaneous breathing was self-attempted. In contrast, there was some neurological function present: spontaneous eye opening and movement. No other motor response was further achieved. His CT scan showed no other abnormalities except from a slight cerebral oedema.

## 3. Discussion

Lazarus phenomenon was first described in the international literature in 1982 and has been followed by a relatively small number of references through the past 30 years, considering the possible underestimation of the incidence [2, 3]. Due to legal concerns, underreporting of these cases is strongly suspected [3, 5]. Previous reports have tried to associate this phenomenon with various mechanisms or coincidences. More specifically, drug abuse, such as opioids and cocaine, has been associated with the phenomenon [6].

 Hyperkalemia caused by renal failure might be related to the pathogenesis of CA and ROSC [7]. However, in other cases, authors support that increased venous return, after discontinuation of mechanical ventilation, might be responsible for the spontaneous restoration of circulation [8]. In addition to previous mechanisms, the increased total dose or delayed effect of adrenaline is possibly related [9]. Moreover, in a significant number of cases, the phenomenon occurred in an operating theatre, where blood gases analysis, mechanical ventilation, or drugs are easily available; furthermore, monitoring is obligatory and not immediately removed from patient [10–12].

In our case, the victim spontaneously restored circulation from asystole, making it one of the most impressive reports of the phenomenon. This incidence occurred in an operating theatre; that is why circulation was detected with invasive BP measurement. The patient had also received an increased total dose of adrenaline (12 mg) during his prolonged CPR. His obesity was also responsible for autopositive end-expiratory pressure (PEEP) during mechanical ventilation impairing thus venous return, a factor that was reversed with discontinuation of mechanical ventilation. Moreover, acute renal failure was present in our patient and was combined with slight hyperkalemia in ROSC. Our patient's course of hospitalization was not different from cases described in the existing literature. Perhaps, one would claim that ROSC occurred after discontinuation of CPR, when fluid resuscitation was adequate. This is excluded from authors as an explanation, as efficacy of fluid resuscitation had been established by both physical and laboratory examination during CPR. Furthermore, the underlying rhythm was asystole.

What is obvious about our patient is that hypovolaemia resulted in CA. Moreover, if the hypovolaemia had not been reversed, no ROSC would have been achieved. The question remaining is what is the factor that needs to be activated or withdrawn, in order to permit spontaneous restoration of circulation? However, 10 min of monitoring after discontinuation of CPR is essential, in order to observe and study more this phenomenon.

## Figures and Tables

**Table 1:** Blood gases and electrolytes' concentration at baseline, various phases of Cardiopulmonary Resuscitation and after Return of Spontaneous Circulation.

	PO_2_ (mmHg)	PCO_2_ (mmHg)	pH	Haemoglobin (mg/dL)	Na^+^ (mEq/L)	K^+^ (mEq/L)
Baseline	120	40	7.32	6.3	135	3.7
1st min of CPR	102	17	7.25	5.2	134	3.2
12th min of CPR	95	12	7.18	7.1	133	4.1
24th min of CPR	92	11	7.10	9.6	136	4.9
45th min of CPR	82	8	7.05	10.2	137	6
ROSC	94	29	7.12	10.1	137	6

CPR: cardiopulmonary resuscitation, ROSC: return of spontaneous circulation.
